# Sustained Progression-Free Survival Benefit of Rituximab Maintenance in Patients With Follicular Lymphoma: Long-Term Results of the PRIMA Study

**DOI:** 10.1200/JCO.19.01073

**Published:** 2019-07-24

**Authors:** Emmanuel Bachy, John F. Seymour, Pierre Feugier, Fritz Offner, Armando López-Guillermo, David Belada, Luc Xerri, John V. Catalano, Pauline Brice, François Lemonnier, Alejandro Martin, Olivier Casasnovas, Lars M. Pedersen, Véronique Dorvaux, David Simpson, Sirpa Leppa, Jean Gabarre, Maria G. da Silva, Sylvie Glaisner, Loic Ysebaert, Anne Vekhoff, Tanin Intragumtornchai, Steven Le Gouill, Andrew Lister, Jane A. Estell, Gustavo Milone, Anne Sonet, Jonathan Farhi, Harald Zeuner, Hervé Tilly, Gilles Salles

**Affiliations:** ^1^ Hospices Civils de Lyon, Université Claude Bernard Lyon 1, Institut National de la Santé et de la Recherche Médicale (INSERM) 1052, Pierre-Bénite, France; ^2^ Royal Melbourne Hospital and University of Melbourne, Melbourne, Victoria, Australia; ^3^ Centre Hospitalier Régional Universitaire de Nancy, Université de Lorraine, INSERM 1256, Nancy, France; ^4^ Ghent University, Ghent, Belgium; ^5^ Hospital Clinic, Barcelona, Spain; ^6^ Charles University, Hradec Králové, Czech Republic; ^7^ Institut Paoli-Calmettes, Aix-Marseille Université, Marseille, France; ^8^ Frankston Hospital and Monash University, Frankston, Victoria, Australia; ^9^ Hôpital Saint-Louis, Assistance Publique–Hôpitaux de Paris, Paris, France; ^10^ Hôpitaux Universitaires Henri Mondor, Université Paris-Est Créteil, INSERM U955, Créteil, France; ^11^ Hospital Universitario de Salamanca–Institute for Biomedical Research of Salamanca, Centro de Investigación Biomédica en Red de Cáncer, Salamanca, Spain; ^12^ Department of Haematology and INSERM 1231, University Hospital F. Mitterrand, Dijon, France; ^13^ Herlev University Hospital, Copenhagen, Denmark; ^14^ Hôpital de Mercy Centre Hospitalier Régional Metz-Thionville, Metz, France; ^15^ North Shore Hospital, Auckland, New Zealand; ^16^ Helsinki University Hospital, University of Helsinki, Helsinki, Finland; ^17^ Hôpital Pitié-Salpêtrière, Paris, France; ^18^ Portuguese Institute of Oncology, Lisbon, Portugal; ^19^ Institut Curie–Hôpital Rene Huguenin, Saint-Cloud, France; ^20^ Institut Universitaire du Cancer de Toulouse–Oncopole, Toulouse, France; ^21^ Saint Antoine Hospital, Assistance Publique–Hôpitaux de Paris, Paris, France; ^22^ Chulalongkorn University, Bangkok, Thailand; ^23^ Centre Hospitalier Universitaire de Nantes, Centre de Recherche en Cancérologie et Immunologie Nantes Angers, INSERM, Université de Nantes, Nantes, France; ^24^ Queen Mary University of London, London, United Kingdom; ^25^ Concord Hospital, Concord, University of Sydney, New South Wales, Australia; ^26^ Fundaleu, Buenos Aires, Argentina; ^27^ UCL, Mont-Godinne, Yvoir, Belgium; ^28^ Centre Hospitalier Universitaire d’Angers, Angers, France; ^29^ F Hoffman-La Roche, Basel, Switzerland; ^30^ Centre Henri-Becquerel, Rouen, France

## Abstract

**PURPOSE:**

The PRIMA study (ClinicalTrials.gov identifier: NCT00140582) established that 2 years of rituximab maintenance after first-line immunochemotherapy significantly improved progression-free survival (PFS) in patients with follicular lymphoma compared with observation. Here, we report the final PFS and overall survival (OS) results from the PRIMA study after 9 years of follow-up and provide a final overview of safety.

**METHODS:**

Patients (> 18 years of age) with previously untreated high–tumor-burden follicular lymphoma were nonrandomly assigned to receive one of three immunochemotherapy induction regimens. Responding patients were randomly assigned (stratified by induction regimen, response to induction treatment, treatment center, and geographic region) 1:1 to receive 2 years of rituximab maintenance (375 mg/m^2^, once every 8 weeks), starting 8 weeks after the last induction treatment, or observation (no additional treatment). All patients in the extended follow-up provided their written informed consent (data cutoff: December 31, 2016).

**RESULTS:**

In total, 1,018 patients completed induction treatment and were randomly assigned to rituximab maintenance (n = 505) or observation (n = 513). Consent for the extended follow-up was provided by 607 patients (59.6%) of 1,018 (rituximab maintenance, n = 309; observation, n = 298). After data cutoff, median PFS was 10.5 years in the rituximab maintenance arm compared with 4.1 years in the observation arm (hazard ratio, 0.61; 95% CI, 0.52 to 0.73; *P* < .001). No OS difference was seen in patients randomly assigned to rituximab maintenance or observation (hazard ratio, 1.04; 95% CI, 0.77 to 1.40; *P* = .7948); 10-year OS estimates were approximately 80% in both study arms. No new safety signals were observed.

**CONCLUSION:**

Rituximab maintenance after induction immunochemotherapy provides a significant long-term PFS, but not OS, benefit over observation.

## INTRODUCTION

Follicular lymphoma (FL) is the second most common lymphoma subtype in the United States and Western Europe, accounting for approximately 25% of all non-Hodgkin lymphoma cases and 70% of indolent lymphomas.^1-3^ Although the prognosis of patients with FL has significantly improved since the introduction of rituximab to first-line (1L) and salvage therapies,^4-10^ advanced-stage FL is believed to remain incurable in most patients because of inevitable relapses; however, strides have been made to prolong the duration of remission without exposure to additional cytotoxic treatment.

Previous studies have demonstrated a significant clinical benefit for rituximab maintenance in patients with relapsed disease after induction with chemotherapy with or without rituximab^9-11^ or single-agent rituximab,^12,13^ and in patients undergoing autologous stem-cell transplantation.^14^ Rituximab maintenance after chemotherapy^15^ or single-agent rituximab^16^ has also been studied in patients with previously untreated FL, with favorable results; however, neither of these induction regimens is considered optimal for patients with advanced-stage disease.

The pivotal PRIMA study (ClinicalTrials.gov identifier: NCT00140582) was the first phase III trial, to our knowledge, to evaluate the potential benefit of 2 years of rituximab maintenance in patients with high–tumor-burden FL responding to 1L rituximab-containing immunochemotherapy.^17^ After a median follow-up of 3 years, rituximab maintenance significantly prolonged progression-free survival (PFS) compared with observation; risk of disease progression was reduced by 45% (hazard ratio [HR], 0.55; 95% CI, 0.44 to 0.68; *P* < .001), and 3-year PFS rates were 74.9% and 57.6%, respectively. This PFS benefit was achieved regardless of the induction regimen, response to induction treatment, or patient age. Time to next antilymphoma treatment (TTNLT) and time to next chemotherapy treatment (TTNCT) were also significantly prolonged with rituximab maintenance, but no overall survival (OS) benefit was seen. An updated 6-year follow-up of the PRIMA study confirmed these results.^18^ Rituximab maintenance is now widely recommended for patients with FL responding to 1L rituximab-based immunochemotherapy.^19^ We present the final PFS and OS results from the PRIMA study after 9 years of follow-up and a final overview of safety.

## METHODS

### Study Design, Patients, and Treatments

PRIMA was an open-label, international, multicenter, randomized phase III trial in patients with previously untreated, high–tumor-burden FL. The study comprised two phases: induction and maintenance or observation (undertaken between December 2004 and April 2007, in 223 centers in 25 countries). Patients eligible for induction therapy were older than 18 years with untreated FL (histologic grade 1, 2, or 3a), diagnosed by a lymph node biopsy performed within 4 months of study registration. Inclusion and exclusion criteria are described in full elsewhere.^17^

During the induction phase, patients received rituximab in combination with cyclophosphamide, doxorubicin, vincristine, and prednisone (CHOP; six cycles); cyclophosphamide, vincristine, and prednisone (CVP; eight cycles); or fludarabine, cyclophosphamide, and mitoxantrone (FCM; six cycles).^17^ Each center selected their preferred regimen for all patients enrolled at that center. Rituximab (375 mg/m^2^) was administered intravenously on day 1 of each chemotherapy course. CHOP- and FCM-treated patients received two additional rituximab infusions to ensure equivalent exposure during the induction phase.

Response was assessed 2 to 4 weeks after last induction treatment. Patients achieving a complete response (CR), an unconfirmed complete response (CRu), or a partial response (PR) were eligible for the next study phase. Eligible patients must have received at least four cycles of rituximab plus CHOP , six cycles of rituximab plus CVP, or four cycles of rituximab plus FCM. At least six infusions of rituximab were required for each treatment regimen, without a delay of more than 2 weeks between each cycle.

Responding patients were randomly assigned 1:1 to receive rituximab maintenance (375 mg/m^2^, once every 8 weeks), starting 8 weeks after last induction treatment, or observation (no additional treatment). All randomly assigned patients received rituximab maintenance or underwent observation for 2 years or until disease progression, whichever occurred first. The random assignment procedure has been reported previously.^17^ Patients who completed this phase were initially followed for 3 years (data cutoff: January 31, 2011, per initial protocol) or 5 years (data cutoff: January 31, 2013, per protocol amendment). Patients in this extended follow-up study consented in writing to approximately 2 more years of follow-up (data cutoff: December 31, 2016).

PRIMA was conducted in accordance with the Declaration of Helsinki and Good Clinical Practice guidelines. The study protocol and amendments were approved by local and national ethics committees, according to the laws of each country. Patients provided written informed consent.

### Assessments

Response was evaluated according to the 1999 International Working Group response criteria for non-Hodgkin lymphoma.^20^ During the 2-year rituximab maintenance or observation phase, patients were assessed by clinical examination every 8 weeks and had a computed tomography scan every 6 months. If bone marrow involvement was initially documented, a biopsy was required at the end-of-treatment assessment to confirm CR. Patients completing the rituximab maintenance or observation phase underwent a final restaging assessment within 28 days of the last rituximab dose (or within a corresponding timeframe for those randomly assigned to observation). For patients with no disease progression, follow-up assessments were scheduled every 3 months for the first 2 years, then every 6 months for an additional 3 years, and then annually in patients consenting to the extended follow-up. Patients with disease progression were followed annually for the initiation of new treatment and OS for 5 years, or until data cutoff in patients consenting to the extended follow-up.

### Efficacy and Safety Analyses

The primary end point was investigator-assessed PFS. Secondary end points included TTNLT, TTNCT, OS, and transformation rate at relapse. Safety outcome measures included adverse events (AEs), serious AEs, grade 3 or higher AEs, and deaths. Grading of AEs was according to National Cancer Institute Common Terminology Criteria for Adverse Events, version 3.0.

### Statistical Analysis

PFS was defined as the time from random assignment to progression, relapse, or death from any cause. Responding patients and patients lost to follow-up were censored at their last tumor assessment date. OS was determined from the date of random assignment to the date of death regardless of cause. Survival end points were estimated by Kaplan-Meier methodology and compared using a two-sided log-rank test stratified by induction regimen and induction response. Histologic transformation rates at first relapse were compared using a χ^2^ test.

## RESULTS

### Patients

**FIG 1.**
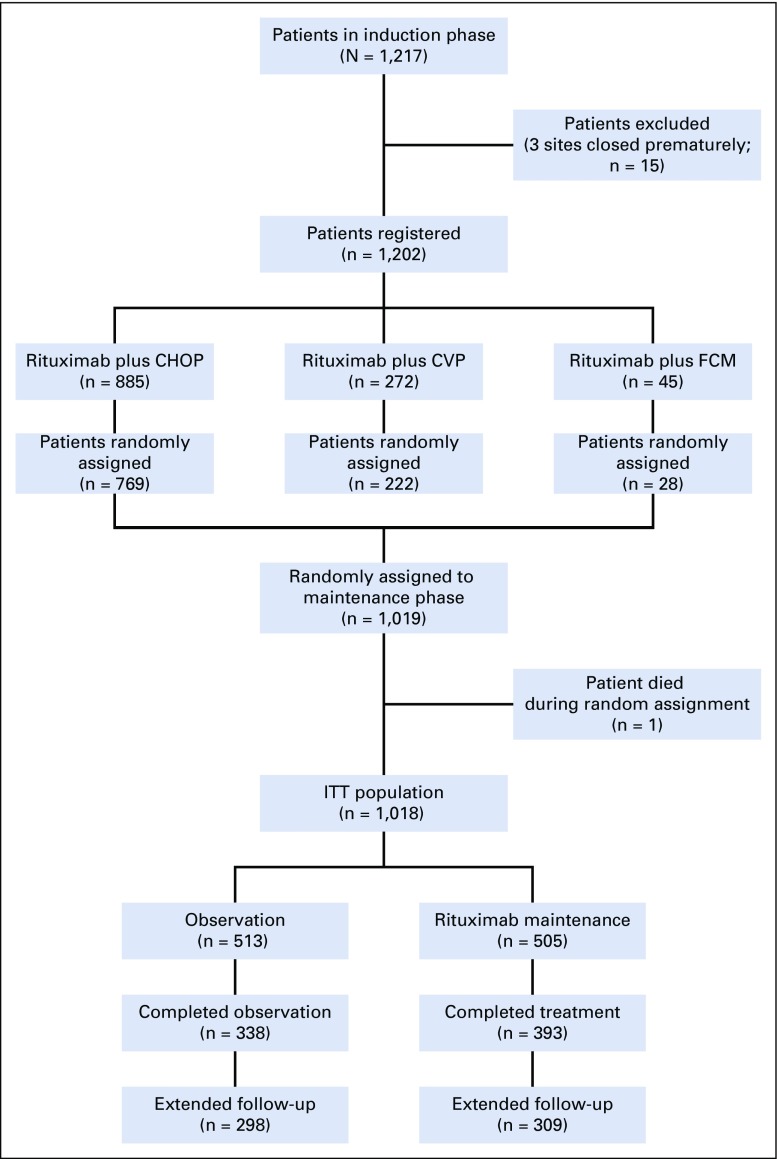
CONSORT diagram. Full details of the trial profile before follow-up have been published previously.^17^ CHOP, cyclophosphamide, doxorubicin, vincristine, and prednisone; CVP, cyclophosphamide, vincristine, and prednisone; FCM, fludarabine, cyclophosphamide, and mitoxantrone; ITT, intent-to-treat.

Overall, 1,018 patients completed induction treatment and were randomly assigned to rituximab maintenance (n = 505) or observation (n = 513); these patients were the primary population for efficacy analyses. Nine patients (rituximab maintenance, n = 4; observation, n = 5) withdrew before the first maintenance treatment cycle or observation visit and were excluded from the safety analyses. Consent for the extended follow-up was provided by 607 patients (59.6%) of 1,018 (rituximab maintenance, n = 309; observation, n = 298). An overview of the trial profile is provided in Figure 1.

Median duration of follow-up was 9.0 years (range, 0.0 to 11.5 years) from random assignment and was well balanced between arms (rituximab maintenance, 9.1 years; observation, 9.0 years). Patient demographics and disease characteristics at random assignment are listed in Table 1. Patients not included in the extended follow-up exhibited adverse prognostic factors more frequently than those in the extended follow-up, mainly because of the automatic exclusion of patients who died before the current analysis [data not shown]).

**TABLE 1.**
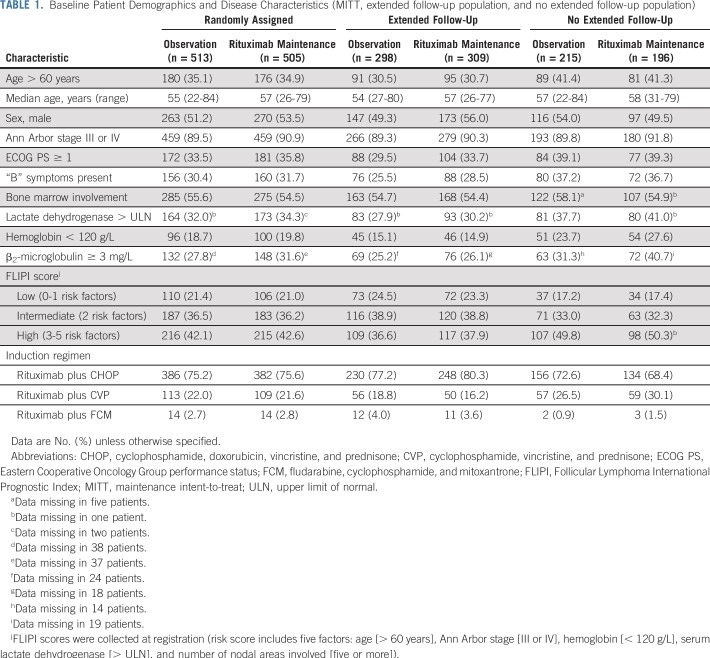
Baseline Patient Demographics and Disease Characteristics (MITT, extended follow-up population, and no extended follow-up population)

### Efficacy

**FIG 2.**
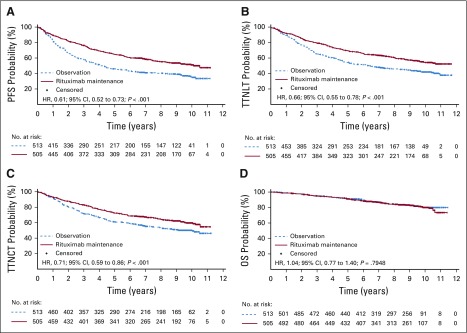
Kaplan-Meier estimates of (A) progression-free survival (PFS), (B) time to next antilymphoma treatment (TTNLT), (C) time to next chemotherapy treatment (TTNCT), and (D) overall survival (OS) from random assignment. HR, hazard ratio.

**FIG 3.**
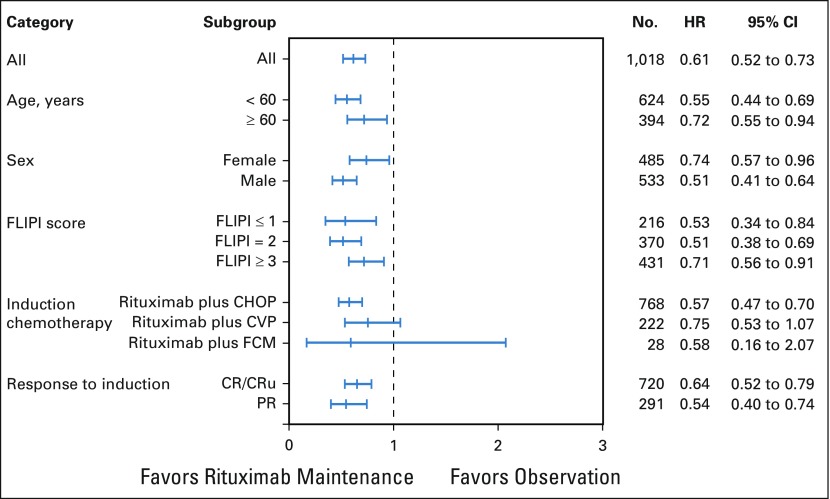
Risk of progression according to prespecified subgroups. CHOP, cyclophosphamide, doxorubicin, vincristine, and prednisone; CR, complete response; CVP, cyclophosphamide, vincristine, and prednisone; FCM, fludarabine, cyclophosphamide, and mitoxantrone; FLIPI, Follicular Lymphoma International Prognostic Index; HR, hazard ratio; PR, partial response; CRu, unconfirmed complete response.

Rituximab maintenance after rituximab-containing induction immunochemotherapy continued to provide a significant long-term PFS benefit compared with observation (Table 2; Fig 2A). At the final data cutoff, median PFS was 10.5 years in patients randomly assigned to rituximab maintenance versus 4.1 years in patients randomly assigned to observation (HR, 0.61; 95% CI, 0.52 to 0.73; *P* < .001). Ten-year PFS estimates were 51.1% in the rituximab maintenance arm and 35.0% in the observation arm. Evaluation of PFS in prespecified patient subgroups, categorized by age, sex, FLIPI score, induction chemotherapy, and response to induction, showed a consistent benefit of rituximab maintenance over observation (Fig 3). Patients in CR, CRu, or PR at end of induction consistently benefited from rituximab maintenance (Data Supplement). PFS by FLIPI risk factor category in the two treatment arms is shown in the Data Supplement.

**TABLE 2.**
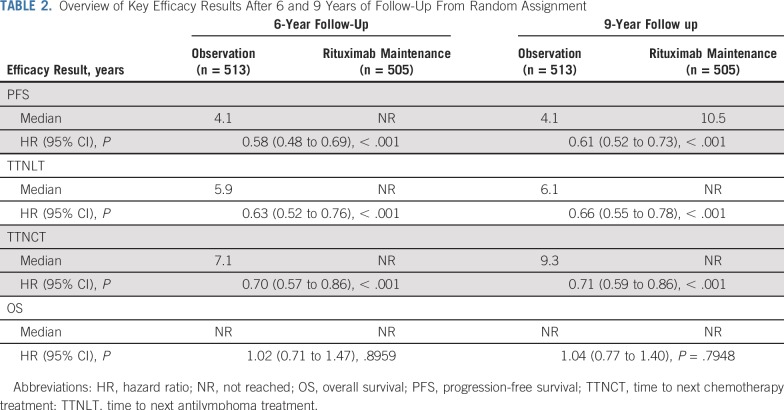
Overview of Key Efficacy Results After 6 and 9 Years of Follow-Up From Random Assignment

Rituximab maintenance also provided a significant benefit over observation in terms of time to next treatment; median TTNLT was not reached in the rituximab maintenance arm versus 6.1 years in the observation arm (HR, 0.66; 95% CI, 0.55 to 0.78; *P* < .001; Table 2; Fig 2B). At the final data cutoff, 212 patients (42.0%) of 505 in the rituximab maintenance arm and 284 patients (55.4%) of 513 in the observation arm had either started a new antilymphoma treatment or died before receiving it. Ten-year TTNLT estimates were 53.4% in the rituximab maintenance arm and 41.2% in the observation arm.

Median TTNCT was not reached in the rituximab maintenance arm versus 9.3 years in the observation arm (HR, 0.71; 95% CI, 0.59 to 0.86; *P* < .001; Table 2; Fig 2C); at data cutoff, 188 patients (37.2%) of 505 in the rituximab maintenance arm and 244 patients (47.6%) of 513 in the observation arm had either started a new chemotherapy treatment or had died before receiving it.

The above-mentioned beneficial effects of rituximab maintenance did not translate into an OS benefit (Table 2; Fig 2D), with 10-year OS rate estimates of approximately 80% (observation, 79.9%; rituximab maintenance, 80.1%) in both study arms; median OS was not reached in either arm (HR, 1.04; 95% CI, 0.77 to 1.40; *P* = .7948). OS according to FLIPI risk factor categories in patients randomly assigned to rituximab maintenance or observation is shown in the Data Supplement. OS after progression or relapse (ie, time from first progression until death) was shorter in the maintenance arm versus the observation arm, explaining equivalent OS in both arms (Data Supplement).

A total of 503 patients had documented disease progression. The rate of progression with disease transformation was low, but similar in both study arms (Data Supplement). No difference in time to transformation was observed (Data Supplement).

### Second-Line Treatment

Of 503 patients who experienced disease progression, 453 received documented second-line (2L) therapy. The most common subsequent chemotherapy regimens were rituximab with a platinum-based regimen (27.2%), fludarabine-based regimen (12.1%), or bendamustine (8.6%; Data Supplement). Significantly more patients in the observation arm than in the rituximab maintenance arm received a rituximab-containing therapy at relapse or progression (81.5% *v* 73.2%, respectively; *P* = .04). Slightly more patients in the observation arm received radioimmunotherapy (24.4% *v* 16.9%, respectively; *P* = .06). One hundred twenty patients (26.5%) underwent high-dose therapy followed by autologous stem-cell transplantation with no difference between the two arms (29.3% *v* 22.4%, respectively; *P* = .13). Response to 2L regimen was similar between 1L treatment arms, with overall response and CR rates of 78.2% and 47.3%, respectively (rituximab maintenance) versus 80.4% and 46.4%, respectively (observation). However, the rate of CR/CRu for patients who experienced early progression within 18 months of random assignment (corresponding to 24 months after induction) was inferior in the maintenance arm compared with the observation arm (39.3% *v* 56.3%; *P* = 0.029), thus demonstrating that the patients who experienced disease progression during maintenance were those with a more aggressive disease (Data Supplement).

### Safety

Since random assignment, 285 patients (56.9%) of 501 in the rituximab maintenance arm and 194 patients (38.2%) of 508 in the observation arm have experienced at least one AE (Data Supplement). Rituximab maintenance was associated with a higher rate of grade 3 to 4 AEs (24.4% *v* 16.9%) and serious AEs (21.2% *v* 13.4%) compared with observation; the higher rate of grade 3 to 4 AEs was driven largely by higher rates of cytopenias (5.2% *v* 1.6%) and infections (4.4% *v* 1.0%). The most common grade 3 to 4 AEs were neoplasms benign, malignant, and unspecified (including cysts and polyps), with a similar incidence between study arms (approximately 4% in both arms). Grade 5 (fatal) AEs occurred in eight patients (1.6%) of 501 and three patients (0.6%) of 508 randomly assigned to rituximab maintenance and observation, respectively (Data Supplement).

A total of 88 patients (17.4%) have died in the rituximab maintenance arm since random assignment versus 84 (16.4%) in the observation arm (Table 3). The most frequent causes of death were progressive disease (rituximab maintenance, 51.1%; observation, 47.6%) and solid tumors (rituximab maintenance, 5.7%; observation, 20.2%).

**TABLE 3.**
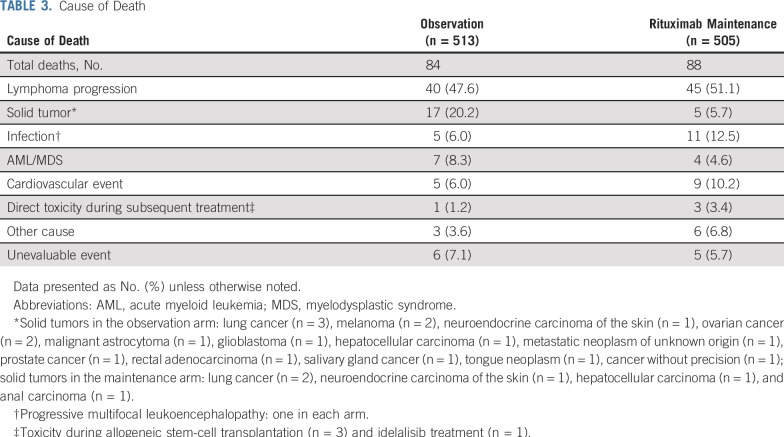
Cause of Death

## DISCUSSION

The primary analysis from PRIMA demonstrated prolonged PFS with rituximab maintenance when applied after 1L immunochemotherapy induction to patients with previously untreated, high–tumor-burden FL.^17^ This long-term follow-up strengthens these previously published results,^17,18^ demonstrating significantly longer PFS, TTNLT, and TTNCT in the rituximab maintenance arm compared with the observation arm. With an updated 9-year median follow-up, projected 10-year PFS was 51.1% in the rituximab maintenance arm and 35.0% in the observation arm, whereas 10-year TTNLT estimates were 53.4% and 41.2%, respectively. Clinically, these results mean that approximately half of patients who receive rituximab maintenance every 8 weeks for 2 years after response to immunochemotherapy induction will remain free from progression or death and free from further antilymphoma treatment after 10 years. Subgroup analyses showed the substantial PFS improvement associated with rituximab maintenance was independent of age, sex, induction immunochemotherapy regimen, response to induction (CR/CRu, or PR), or FLIPI risk score.

The OS estimate at 10 years for the whole patient cohort was approximately 80%, thus confirming how the introduction of rituximab into the therapeutic armamentarium in general, and into the 1L induction setting in particular, has profoundly altered the course of FL, as compared with historical controls.^21,22^ However, despite significant and sustained PFS prolongation with rituximab maintenance, no OS difference was observed between the two arms. This finding is similar to recently published long-term follow-up studies in which prolonged PFS with the use of rituximab plus CHOP (compared with rituximab plus CVP) or rituximab plus bendamustine (compared with rituximab plus CHOP) fails to translate into prolonged OS^23,24^ and has important implications for both our understanding of the disease and future research in the field. First, direct extrapolation of PFS as a surrogate marker for OS cannot be made in FL, even with long-term follow-up. Second, PFS and TTNLT prolongation as meaningful clinical and economic end points must be viewed independently of OS. And third, the underlying biologic explanation for PFS improvement not translating into longer OS needs to be addressed.

Recent efforts have been made by the Follicular Lymphoma Analysis of Surrogacy Hypothesis) group to assess if CR at 30 months after initiation of induction therapy can serve as a surrogate end point for PFS in FL, and the initial results look promising.^25^ However, the evidence for PFS as a surrogate for OS is conflicting. In advanced solid tumors, there is considerable heterogeneity among cancer types and, for a given neoplasm, there are even discrepancies among the same histology subgroups, resulting in a generally low strength of association between PFS and OS.^26^ In lymphoma, surrogacy has been studied and documented in 1L diffuse large B-cell lymphoma,^27^ but robust data are lacking in FL. Indeed, statistical modeling indicates that the association between PFS and OS tends to be weaker for malignancies with a long survival after progression, such as FL, which explains how the PFS advantage reported here may have been diluted over subsequent lines of treatment.^28^ Whether the recently described progression of disease within 24 months of initiating treatment end point is a more reliable surrogate of OS in patients with FL receiving 1L immunochemotherapy with or without maintenance remains to be established.^29-32^

In our analysis, the proportion of deaths associated with lymphoma progression was almost identical between treatment arms. Response to 2L treatment was also comparable. Shorter survival after first relapse in the maintenance arm helps to explain why OS was similar in both arms despite prolonged PFS. Altogether, these data indicate that rituximab maintenance does not alter the natural course of the disease for patients with aggressive FL and that they will ultimately die as rapidly as if they were observed after induction treatment. Whether the absence of an OS benefit in these patients challenges the appeal of a prolonged 1L remission in most patients with de novo FL with a high tumor burden remains an open question.

No difference in terms of transformation rate was found with this extended follow-up, and these findings confirmed a previous analysis of data from the PRIMA cohort, which showed that rituximab maintenance did not have a significant prognostic impact on histologic transformation.^33^ Interestingly, detailed analysis of 2L treatments at relapse showed that use of rituximab was significantly less frequent after rituximab maintenance than after observation. Data on the use of anti-CD20 antibodies as maintenance at relapse were lacking, but one could hypothesize that rituximab maintenance may have been more frequently administered in the observation arm, given the established beneficial effect of rituximab maintenance on PFS in the relapsed/resistant setting.^34^ This could potentially explain, at least in part, the absence of a difference in OS between the two arms.

Consistent with previous analyses,^17,18^ rituximab maintenance was generally well tolerated, and no unexpected safety signals were observed with the additional 4 years of follow-up. It is worth noting that although the OS rate was not different between the two arms, death due to second neoplasia was almost four times more frequent in the observation arm compared with the maintenance arm. It could be speculated that recurrent use of cytotoxic- and radiation-containing regimens in the context of earlier relapse in the observation arm may have increased the frequency of second neoplasms. Conversely, deaths due to infection, a known consequence of immunotherapy,^34^ were twice as frequent in the rituximab maintenance arm. However, only two cases of the opportunistic infection, progressive multifocal leukoencephalopathy, were observed, one in each treatment arm. Although rituximab exposure may increase this risk,^35^ our data suggest there is not a strong effect of maintenance.

In conclusion, this 9-year follow-up of the PRIMA study demonstrates that rituximab maintenance after induction immunochemotherapy provides a significant long-term PFS benefit over observation. Despite the lack of OS advantage, it is noteworthy that more than half of the patients in the rituximab maintenance arm remain free of disease progression and have not required new antilymphoma treatment beyond 10 years.

## References

[1] Freedman A Follicular lymphoma: 2018 update on diagnosis and management Am J Hematol 93 296 305 2018 29314206 10.1002/ajh.24937

[2] Swerdlow SH Campo E Pileri SA et al The 2016 revision of the World Health Organization classification of lymphoid neoplasms Blood 127 2375 2390 2016 26980727 10.1182/blood-2016-01-643569 PMC4874220

[3] A clinical evaluation of the International Lymphoma Study Group classification of non-Hodgkin’s lymphoma The Non-Hodgkin’s Lymphoma Classification Project Blood 89 3909 3918 1997 9166827

[4] Marcus R Imrie K Belch A et al CVP chemotherapy plus rituximab compared with CVP as first-line treatment for advanced follicular lymphoma Blood 105 1417 1423 2005 15494430 10.1182/blood-2004-08-3175

[5] Marcus R Imrie K Solal-Celigny P et al Phase III study of R-CVP compared with cyclophosphamide, vincristine, and prednisone alone in patients with previously untreated advanced follicular lymphoma J Clin Oncol 26 4579 4586 2008 18662969 10.1200/JCO.2007.13.5376

[6] Salles G Mounier N de Guibert S et al Rituximab combined with chemotherapy and interferon in follicular lymphoma patients: Results of the GELA-GOELAMS FL2000 study Blood 112 4824 4831 2008 18799723 10.1182/blood-2008-04-153189

[7] Herold M Haas A Srock S et al Rituximab added to first-line mitoxantrone, chlorambucil, and prednisolone chemotherapy followed by interferon maintenance prolongs survival in patients with advanced follicular lymphoma: An East German Study Group Hematology and Oncology Study J Clin Oncol 25 1986 1992 2007 17420513 10.1200/JCO.2006.06.4618

[8] Hiddemann W Kneba M Dreyling M et al Frontline therapy with rituximab added to the combination of cyclophosphamide, doxorubicin, vincristine, and prednisone (CHOP) significantly improves the outcome for patients with advanced-stage follicular lymphoma compared with therapy with CHOP alone: Results of a prospective randomized study of the German Low-Grade Lymphoma Study Group Blood 106 3725 3732 2005 16123223 10.1182/blood-2005-01-0016

[9] van Oers MH Klasa R Marcus RE et al Rituximab maintenance improves clinical outcome of relapsed/resistant follicular non-Hodgkin lymphoma in patients both with and without rituximab during induction: Results of a prospective randomized phase 3 intergroup trial Blood 108 3295 3301 2006 16873669 10.1182/blood-2006-05-021113

[10] Forstpointner R Unterhalt M Dreyling M et al Maintenance therapy with rituximab leads to a significant prolongation of response duration after salvage therapy with a combination of rituximab, fludarabine, cyclophosphamide, and mitoxantrone (R-FCM) in patients with recurring and refractory follicular and mantle cell lymphomas: Results of a prospective randomized study of the German Low Grade Lymphoma Study Group (GLSG) Blood 108 4003 4008 2006 16946304 10.1182/blood-2006-04-016725

[11] van Oers MH Van Glabbeke M Giurgea L et al Rituximab maintenance treatment of relapsed/resistant follicular non-Hodgkin’s lymphoma: Long-term outcome of the EORTC 20981 phase III randomized intergroup study J Clin Oncol 28 2853 2858 2010 20439641 10.1200/JCO.2009.26.5827 PMC2903319

[12] Ghielmini M Schmitz SF Cogliatti SB et al Prolonged treatment with rituximab in patients with follicular lymphoma significantly increases event-free survival and response duration compared with the standard weekly x 4 schedule Blood 103 4416 4423 2004 14976046 10.1182/blood-2003-10-3411

[13] Hainsworth JD Litchy S Shaffer DW et al Maximizing therapeutic benefit of rituximab: maintenance therapy versus re-treatment at progression in patients with indolent non-Hodgkin’s lymphoma--A randomized phase II trial of the Minnie Pearl Cancer Research Network J Clin Oncol 23 1088 1095 2005 15657401 10.1200/JCO.2005.12.191

[14] Pettengell R Schmitz N Gisselbrecht C et al Rituximab purging and/or maintenance in patients undergoing autologous transplantation for relapsed follicular lymphoma: A prospective randomized trial from the lymphoma working party of the European group for blood and marrow transplantation J Clin Oncol 31 1624 1630 2013 23547078 10.1200/JCO.2012.47.1862

[15] Hochster H Weller E Gascoyne RD et al Maintenance rituximab after cyclophosphamide, vincristine, and prednisone prolongs progression-free survival in advanced indolent lymphoma: Results of the randomized phase III ECOG1496 Study J Clin Oncol 27 1607 1614 2009 19255334 10.1200/JCO.2008.17.1561 PMC2668968

[16] Martinelli G Schmitz SF Utiger U et al Long-term follow-up of patients with follicular lymphoma receiving single-agent rituximab at two different schedules in trial SAKK 35/98 J Clin Oncol 28 4480 4484 2010 20697092 10.1200/JCO.2010.28.4786

[17] Salles G Seymour JF Offner F et al Rituximab maintenance for 2 years in patients with high tumour burden follicular lymphoma responding to rituximab plus chemotherapy (PRIMA): A phase 3, randomised controlled trial Lancet 377 42 51 2011 21176949 10.1016/S0140-6736(10)62175-7

[18] Salles G Seymour JF Feugier P et al Updated 6 year follow-up of the PRIMA study confirms the benefit of 2-year rituximab maintenance in follicular lymphoma patients responding to frontline immunochemotherapy Blood 122 509 2013

[19] Dreyling M Ghielmini M Rule S et al Newly diagnosed and relapsed follicular lymphoma: ESMO Clinical Practice Guidelines for diagnosis, treatment and follow-up Ann Oncol 28 3109 2017 suppl_4 28327933 10.1093/annonc/mdx020

[20] Cheson BD Horning SJ Coiffier B et al Report of an international workshop to standardize response criteria for non-Hodgkin’s lymphomas J Clin Oncol 17 1244 1999 10561185 10.1200/JCO.1999.17.4.1244

[21] Fisher RI LeBlanc M Press OW et al New treatment options have changed the survival of patients with follicular lymphoma J Clin Oncol 23 8447 8452 2005 16230674 10.1200/JCO.2005.03.1674

[22] Liu Q Fayad L Cabanillas F et al Improvement of overall and failure-free survival in stage IV follicular lymphoma: 25 years of treatment experience at The University of Texas M.D. Anderson Cancer Center J Clin Oncol 24 1582 1589 2006 16575009 10.1200/JCO.2005.03.3696

[23] Luminari S Ferrari A Manni M et al Long-term results of the FOLL05 trial comparing R-CVP versus R-CHOP versus R-FM for the initial treatment of patients with advanced-stage symptomatic follicular lymphoma J Clin Oncol 36 689 696 2018 29095677 10.1200/JCO.2017.74.1652

[24] Rummel M Maschmeyer G Ganser A et al Bendamustine plus rituximab (B-R) versus CHOP plus rituximab (CHOP-R) as first-line treatment in patients with indolent lymphomas: Nine-year updated results from the StiL NHL1 study J Clin Oncol 35 2017 15_suppl; abstr 7501

[25] Shi Q Flowers CR Hiddemann W et al Thirty-month complete response as a surrogate end point in first-line follicular lymphoma therapy: An individual patient-level analysis of multiple randomized trials J Clin Oncol 35 552 560 2017 28029309 10.1200/JCO.2016.70.8651

[26] Ciani O Davis S Tappenden P et al Validation of surrogate endpoints in advanced solid tumors: Systematic review of statistical methods, results, and implications for policy makers Int J Technol Assess Health Care 30 312 324 2014 25308694 10.1017/S0266462314000300

[27] Shi Q Schmitz N Ou FS et al Progression-free survival as a surrogate end point for overall survival in first-line diffuse large B-cell lymphoma: An individual patient-level analysis of multiple randomized trials (SEAL) J Clin Oncol 36 2593 2602 2018 29975624 10.1200/JCO.2018.77.9124 PMC6532366

[28] Broglio KR Berry DA Detecting an overall survival benefit that is derived from progression-free survival J Natl Cancer Inst 101 1642 1649 2009 19903805 10.1093/jnci/djp369 PMC4137232

[29] Casulo C Byrtek M Dawson KL et al Early relapse of follicular lymphoma after rituximab plus cyclophosphamide, doxorubicin, vincristine, and prednisone defines patients at high risk for death: An analysis from the National LymphoCare Study J Clin Oncol 33 2516 2522 2015 26124482 10.1200/JCO.2014.59.7534 PMC4879714

[30] Jurinovic V Kridel R Staiger AM et al Clinicogenetic risk models predict early progression of follicular lymphoma after first-line immunochemotherapy Blood 128 1112 1120 2016 27418643 10.1182/blood-2016-05-717355 PMC5457130

[31] Launonen A, Hiddeman W, Duenzinger U, et al: Early disease progression predicts poorer survival in patients with follicular lymphoma (FL) in the GALLIUM study. Blood 130:1490, 2017

[32] Seymour JF Marcus R Davies A et al Association of early disease progression and very poor survival in the GALLIUM study in follicular lymphoma: Benefit of obinutuzumab in reducing the rate of early progression Haematologica 104 1202 1208 2019 30573503 10.3324/haematol.2018.209015 PMC6545851

[33] Sarkozy C Trneny M Xerri L et al Risk factors and outcomes for patients with follicular lymphoma who had histologic transformation after response to first-line immunochemotherapy in the PRIMA trial J Clin Oncol 34 2575 2582 2016 27298402 10.1200/JCO.2015.65.7163

[34] Vidal L Gafter-Gvili A Salles G et al Rituximab maintenance for the treatment of patients with follicular lymphoma: An updated systematic review and meta-analysis of randomized trials J Natl Cancer Inst 103 1799 1806 2011 22021664 10.1093/jnci/djr418

[35] Carson KR Evens AM Richey EA et al Progressive multifocal leukoencephalopathy after rituximab therapy in HIV-negative patients: A report of 57 cases from the Research on Adverse Drug Events and Reports project Blood 113 4834 4840 2009 19264918 10.1182/blood-2008-10-186999 PMC2686134

